# Acute Effects of Cannabinoid Combination Therapies in a Western Diet-Induced Murine Model of Metabolic Liver Disease

**DOI:** 10.3390/ijms27114872

**Published:** 2026-05-28

**Authors:** Jerome Lian, Mohan Patil, Ricky R. Lareu, Marco Falasca

**Affiliations:** 1 Diagnostic and Therapeutic Sciences and Curtin Medical Research Institute, Faculty of Health Sciences, Curtin University, Perth, WA 6102, Australia; jerome.lian@postgrad.curtin.edu.au (J.L.); ricky.lareu@curtin.edu.au (R.R.L.); 2 Molecular Endocrinology and Pharmacology, Harry Perkins Institute of Medical Research and Centre for Medical Research, The University of Western Australia, Perth, WA 6000, Australia; mohan.patil@uwa.edu.au; 3 Department of Medicine and Surgery, University of Parma, Via Volturno 39, 43125 Parma, Italy

**Keywords:** MASH, endocannabinoidome, L-α-lysophosphatidylinositol/G-protein coupled receptor 55, cannabidiol, oleoylethanolamide

## Abstract

Pharmacological treatment of metabolic-dysfunction-associated steatohepatitis remains challenging due to its complex pathophysiology. The endocannabinoidome (eCB) has emerged as a promising therapeutic target given its central role in energy homeostasis and its pharmacological tractability. Western-style diets high in fat and sugar exacerbate metabolic liver disease, highlighting the need for effective interventions. Here, we investigated the therapeutic potential of cannabinoid combinations targeting the eCB–liver axis in a Western diet-induced model of metabolic dysfunction. Two weeks of treatment reduced body weight, improved glycaemic control, and ameliorated liver pathology. These effects were accompanied by decreased liver weight, improved liver enzyme profiles, and reduced histological features of steatosis and injury. Overall, these findings suggest that modulation of the eCB system can induce acute improvements in metabolic and hepatic parameters under conditions of diet-induced metabolic stress. These results support further investigation into the eCB system as a therapeutic target, particularly to elucidate underlying mechanisms and longer-term effects.

## 1. Introduction

Metabolic Dysfunction-Associated Steatotic Liver Disease (MASLD), previously known as Non-Alcoholic Fatty Liver Disease (NAFLD), represents a global public health concern of increasing significance, as it is strongly correlated with obesity, type 2 diabetes mellitus (T2DM), insulin resistance and other cardiometabolic disorders [1,2]. MASLD is now the most prevalent chronic liver condition worldwide, affecting nearly a third of the adult population, with projections indicating a continued rise paralleling the global obesity epidemic [3,4]. MASLD ranges from simple steatosis to more severe liver diseases, including Metabolic Dysfunction-Associated Steatohepatitis (MASH) and hepatocellular carcinoma (HCC), with an increasing risk of liver dysfunction [5,6,7]. Despite its high prevalence, primary interventions for MASH remain lifestyle modifications, including weight loss through diet and exercise, while approved pharmacological treatment options remain limited [8,9].

The endocannabinoidome (eCB) has emerged as an exciting therapeutic target for MASH, owing to its central role in the maintenance of metabolic homeostasis [10]. The eCB is an expansive and intricate physiological system that encompasses the endocannabinoid system (ECS), alongside the broad network of receptors, lipid mediators, enzymes and metabolic pathways that regulate homeostasis [10,11]. Functionally, the eCB acts as a lipid-based signalling system that modulates inter-organ communication and cellular responses to energy demand, nutrient influx, stress and inflammation [12]. Relating to MASH, the L-α-lysophosphatidylinositol/G-protein-coupled receptor 55 (LPI/GPR55) system within the eCB has been found to be a driver behind increased hepatic steatosis [13,14], exacerbating chronic low-grade inflammation and hepatocyte stress [14]. Conversely, G-protein-coupled receptors (GPCRs) such as GPR119 are promising therapeutic targets in MASLD/MASH, due to their role in regulating glucose and lipid metabolism [15].

Cannabidiol (CBD), a non-psychoactive phytocannabinoid derived from *Cannabis sativa*, has attracted growing interest as a potential therapeutic agent in metabolic disorders, largely owing to its modulatory effects on the eCB system [16]. Preclinical studies have demonstrated that CBD attenuates adiposity and reduces inflammatory signalling [17,18,19]. In the gastrointestinal tract, CBD exerts its modulatory role by inhibiting the degradation of the eCB’s endogenous ligands and has also been found to be an antagonist of GPR55 [20]. Notably, CBD is well tolerated in humans [21].

Oleoylethanolamide (OEA) is an endogenous lipid mediator belonging to the N-acylethanolamine family and a natural ligand of GPR119. It is a potent anorectic agent and a key regulator of lipid metabolism [22,23]. Moreover, OEA plays a critical role in energy homeostasis, appetite control, lipid handling, and inflammatory signalling [24,25,26,27]. Primarily, OEA shows high affinity for the peroxisome proliferator-activated receptor alpha (PPAR-α), causing changes to lipid metabolism. OEA activates GPR119, but its anorectic effects are mediated via PPAR-α and transient receptor potential vanilloid 1 (TRPV1) [25,26,28,29].

Previously, we reported that the lipid oleoyl-lysophosphatidylinositol (Oleoyl-LPI) stimulates the secretion of the gut hormone glucagon-like peptide1 (GLP-1) from intestinal L-cells through GPR119 [28]. Oleoyl-LPI occurs at low levels, so we synthesised the mimetic compound PS318 to harness its therapeutic potential. Compound ps318 acts similarly to Oleoyl-LPI, binding to the orthosteric site of GPR119 [29]. The safety and efficacy of compound PS318 have been reported in preclinical studies [30].

In MASH, OEA and CBD modulate GPR55 and GPR119, receptors involved in metabolism and inflammation [31,32]. However, their hepatic roles in disease progression remain unclear and require further study [14,15,28,33]. Pharmacological targeting of the eCB is novel, particularly with emerging roles for LPI/GPR55 and GPR119 in hepatic steatosis [34].

This study evaluated CBD, OEA, and a synthetic oleoyl-LPI mimetic (PS318) as mono- and combination therapies in a Western diet (WD)-induced mouse model of metabolic liver disease, assessing metabolic and hepatic outcomes after 14 days.

## 2. Results

### 2.1. Effect of WD Treatment on Weight and Energy Intake

**Figure 1:**
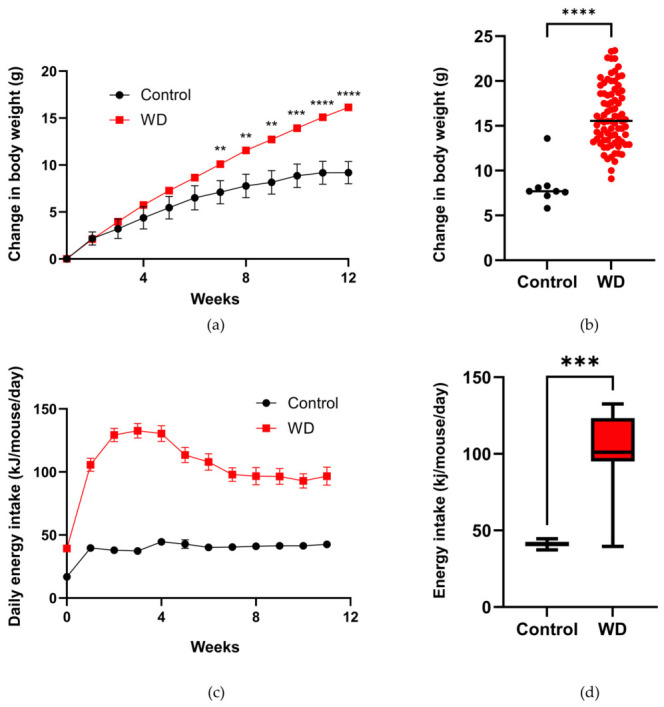
Establishment of disease model over 12 weeks for the Control group (*n* = 10) on normal chow and the group on a Western-style diet (WD) to generate the MASH disease model (*n* = 80). (**a**) Cumulative change in body weight over 12 weeks showing a statistical increase in weight from week 7 for the disease group and (**b**) cumulative change in weight at week 12 showing population distribution with median. (**c**) Energy consumption in kJ/mouse/day over the 12 weeks showing a statistical increase in consumption from week 1 and (**d**) the average energy consumption of mice per week. Statistical analyses of data (see Section 4.6 for details): (**a**,**c**) normally distributed; (**b**,**d**) not normally distributed. Abbreviations: **, *p* < 0.01; ***, *p* < 0.001; ****, *p* < 0.0001; kJ, kilojoules; WD, Western-style fast-food diet.

Twelve weeks of WD feeding resulted in obesity in the C57BL6/J mice, with body weights averaging 38.1 g compared to 28.4 g of C57BL6/J mice fed a normal chow diet (Figure 1a). After the 12-week diet treatment, mice fed a normal chow diet had an average weight gain of 0.75 g ± 0.08 per week, while mice fed a WD showed an average weight gain of 1.37 g ± 0.19 per week (Figure 1a). At week 7 of the diet, cumulative changes in weight for mice on the WD reached statistical significance when compared to mice fed a maintenance diet (*p* < 0.0023, Figure 1b).

Additionally, we also measured the feed intake of WD-treated mice compared to controls. Over the course of 12 weeks, mice fed a maintenance diet ate an average of 3.19 ± 0.17 g of feed per day, while all animals fed a WD consumed 5.19 ± 0.24 g of feed per day (*p* < 0.0315). This feed intake data was then further transformed into energy intake per day (Figure 1c,d); considering the differences in total energy of maintenance diet (12.8 kJ/g) vs. WD (19.7 kJ/g), we see that WD-fed mice consumed more energy on average as compared to mice fed a maintenance diet (102.2 ± 6.97 vs. 40.85 ± 0.64 kJ/day, *p* < 0.001).

### 2.2. Effect of Cannabinoid Treatment on Body Weight and Feed Intake

**Figure 2:**
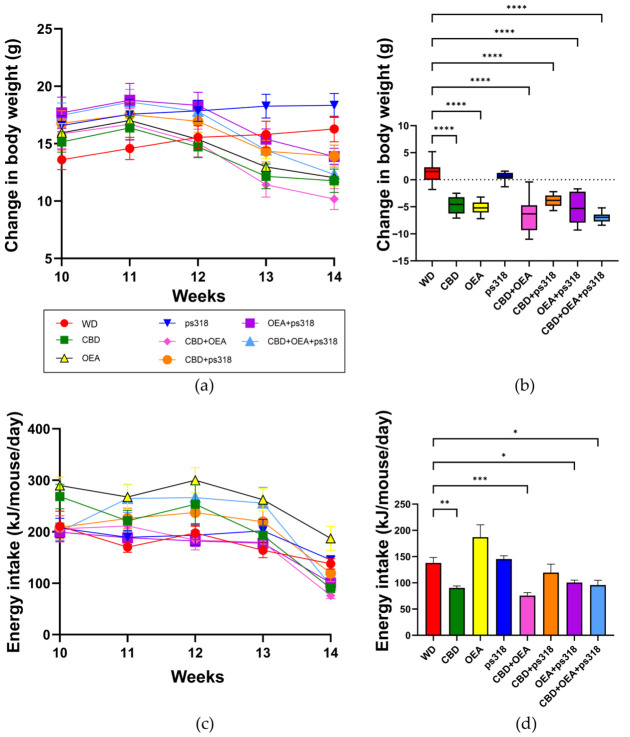

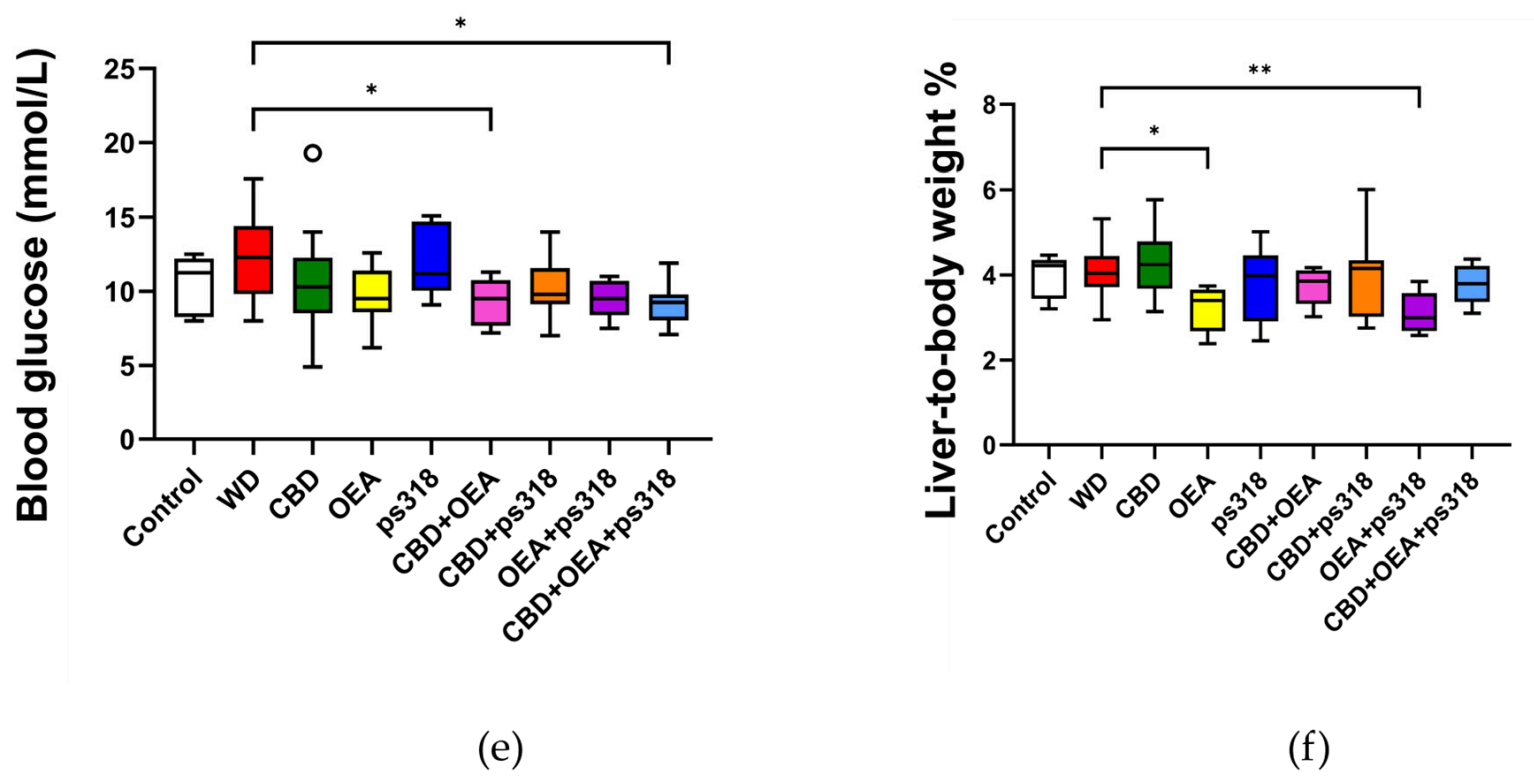
Impact on mouse physiological measures when treated daily for 2 weeks with cannabinoid mono- and combination therapies. (**a**) Cumulative change in body weight between weeks 10 and 14 with treatment starting at week 12. (**b**) Final cumulative change in weight (week 14) showing a statistical reduction in weight compared to the WD group for all treatments except for the ps318 group. (**c**) Cumulative change in energy consumption in kJ/mouse/day between 10 and 14 weeks with a statistical reduction in energy intake at (**d**) week 14 for the CBD, CBD+OEA, OEA+ps318 and the triple combination groups. (**e**) There was a general reduced trend in fasting blood glucose at week 14 with the cannabinoid treatments except for ps318, with CBD+OEA and the triple combination showing statistical significance. (**f**) A statistically significant reduction in liver-to-body weight ratio was seen for the OEA and OEA-ps318 treatments. Statistical analyses of data (see Section 4.7 for details): (**b**,**e**,**f**) normally distributed; (**d**) not normally distributed; 1 outlier removed from the Control group. Statistical analyses were conducted relative to the WD group unless otherwise specified. Abbreviations: *, *p* < 0.05; **, *p* < 0.01; ***, *p* < 0.001; ****, *p* < 0.0001; kJ, kilojoules; WD, Western-style fast-food diet.

Two-week cannabinoid treatments resulted in significant body weight loss in WD-fed mice. Compared to the WD arm, CBD-treated (4.78 g ± 0.49, *p* < 0.0001) and OEA-treated (5.19 ± 0.40, *p* < 0.0001) mice showed significantly reduced body weights (Figure 2a,b). Body weight reduction was further amplified when the agents were administered in combinations. The CBD with OEA combination showed an average weight loss of 6.47 g ± 0.31, while combination therapy involving CBD and compound ps318 showed an average weight loss of 3.90 g ± 0.11. Co-administration of OEA and compound ps318 showed greater weight loss, averaging 5.30 g ± 0.28; however, triple combination treatment involving CBD, OEA and compound ps318 showed the greatest weight loss at 6.99 g ± 0.30.

Changes to average energy intake (Figure 2c,d), representative of the gold standard in treatment for MASH, was also observed in mice treated with cannabinoids. WD mice maintained an average of 59.10 kJ/day. Interestingly, OEA, an anorectic agent, had the opposite effect, showing that these mice consumed on average 80 kJ/day. Treatment with CBD also decreased energy intake, whereby energy intake dropped to 38.70 kJ/day, *p* < 0.0083. Combination treatment of CBD and OEA resulted in a significant decrease in average energy intake at 32.48 kJ/day, *p* < 0.0009, while combination treatment of CBD and compound ps318 showed reduced energy intake to 51.16 kJ/day. Similarly, co-therapy of OEA and compound ps318 decreased energy intake to 43.11 kJ/day. Unsurprisingly, the triple combination of CBD, OEA and compound ps318 also decreased energy intake to 41 kJ/day, although this was not statistically significant.

C57BL6/J mice fed a WD developed hyperglycaemia as expected (Figure 2e). Compared to controls, WD significantly increased fasting blood glucose levels (10.4 ± 0.59 vs. 12.16 ± 0.98 mmol/L, *p* < 0.0298). Compared to WD, monotherapy of CBD reduced fasting blood glucose (10.71 ± 1.231 mmol/L), while OEA alone also decreased blood glucose (9.78 ± 0.61 mmol/L). Combination treatment of CBD with OEA showed a significant reduction in average fasting blood glucose levels (9.33 ± 0.48 mmol/L, *p* < 0.0401). Co-therapy of CBD with ps318 also non-significantly reduced average fasting blood glucose levels (10.27 ± 0.63 mmol/L,), as did OEA in combination with ps318 (9.43 ± 0.37 mmol/L). Combination treatment involving all three cannabinoids: CBD, OEA and ps318 showed the greatest reduction in average fasting blood glucose (9.16 ± 0.42 mmol/L, *p* < 0.0256).

### 2.3. Effect on Liver Condition

#### 2.3.1. Effect on Liver Weight

Chronic WD feeding in mice increased the liver-to-body weight ratio, a critical morphometric index in metabolic liver disease [35] (Figure 2f). Control mice fed a normal chow diet had an average of 0.039 g liver/g body weight, while mice fed the WD had an average of 0.042 g liver/g body weight. Interestingly, CBD did not decrease the liver-to-body weight ratio (0.043 g liver/g body weight), despite causing significant body weight loss. OEA significantly decreased liver-to-body weight ratio (0.032 g, *p* < 0.0195) while compound ps318 also non-significantly reduced the liver-to-body weight ratio to 0.038 g liver/g body weight. However, in combination, both OEA and compound ps318 significantly (*p* < 0.0082) reduce the liver-to-body weight ratio to 0.031 g liver/g body weight. CBD seemed to negate any previous improvements, as CBD in combination with OEA did not significantly reduce liver-to-body weight ratio (0.037 g liver/g body weight). Similarly, CBD in co-therapy with compound ps318 (0.040 g liver/g body weight) or in triple combination with OEA and compound ps318 (0.038 g liver/g body weight) had no statistically significant reductions in the liver-to-body weight ratios.

#### 2.3.2. Effect on Hepatic Steatosis, Ballooning and Inflammation

**Figure 3:**
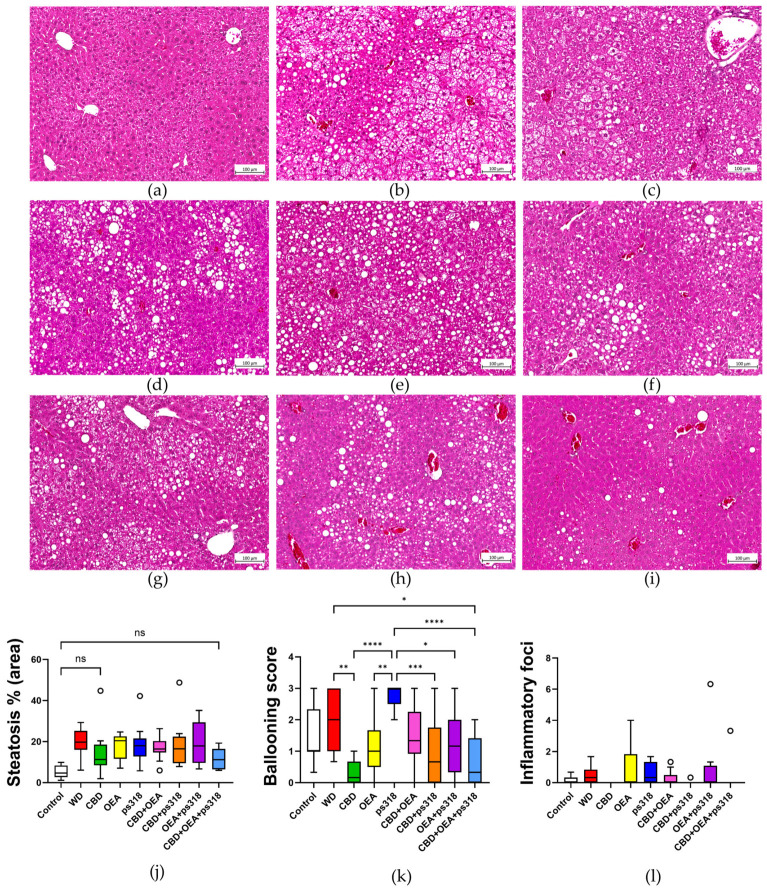
Liver histopathology following cannabinoid treatments. Representative H&E-stained sections for each of the treatments: (**a**) Control; (**b**) WD; (**c**) CBD; (**d**) OEA; (**e**) ps318; (**f**) CBD+OEA; (**g**) CBD+ps318; (**h**) OEA+ps318; and (**i**) CBD+OEA+ps318. (**j**) Total steatosis percentage area was quantified and all treatment groups except for CBD and the triple combination were statistically significant to the Control group. (**k**) For hepatocyte ballooning, only the CBD and the triple combination had scores that were statistically significantly different from the WD group. (**i**) Number of inflammation foci. Circle points indicate outliers which were not included in statistical analysis. All data was analysed through non-parametric analysis. Statistical analyses of data (see Section 4.7 for details): (**k**) data was normally distributed; 2 outliers were removed from the CBD data and 1 from the ps318 data; (**j**,**l**) were not normally distributed. Statistical comparisons were performed relative to the WD group unless otherwise specified. Abbreviations: ns, not significant; *, *p* < 0.05; **, *p* < 0.01; ***, *p* < 0.001; ****, *p* < 0.0001; WD, Western-style fast-food diet.

Hepatic steatosis, the defining histopathological feature of MASLD, is characterised by the abnormal accumulation of triglyceride-rich lipid droplets within hepatocytes [36]. Image analysis of H&E-stained liver tissue sections indicated that in the WD group, there was steatosis observed in 5–33% of hepatocytes (Figure 3a–j). When compared to healthy controls, we saw that the CBD and triple combination treatment groups were not statistically significant, indicating similar levels of steatosis. Furthermore, the development of MASH using WD typically sees a high amount of hepatocyte damage, in the form of hepatic ballooning (Figure 3k). Hepatocellular ballooning is a hallmark feature of progressive steatohepatitis [37]. In the current study, we observed that combination treatment using cannabinoids attenuated hepatic ballooning, with the CBD treatment group significantly reducing ballooning scores (*p* < 0.0012). Triple combination treatment also significantly (*p* < 0.0085) reduced ballooning scores compared to WD. Furthermore, examination of stained liver tissues indicated that CBD alone, the CBD and ps318 combination and triple combination treatment groups reduced the number of inflammatory foci (Figure 3l).

**Figure 4:**
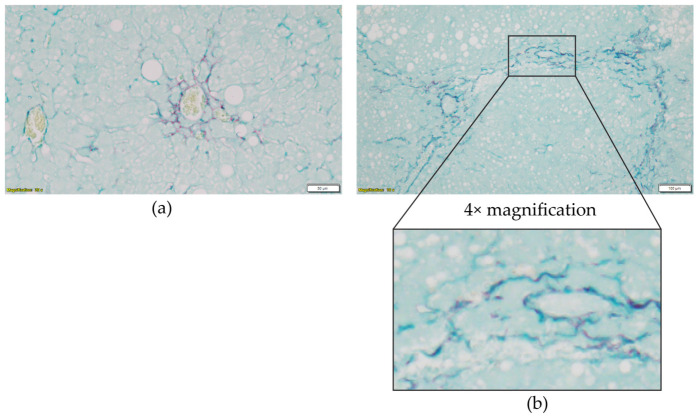
Liver fibrosis. Representative images stained with picrosirius red for collagen and counter stain fast green for other structures (i.e., nucleus and cytoplasm). Red stains indicate fibrosis. (**a**) F1, pericellular fibrosis around a central vein; (**b**) F3, bridging fibrosis; magnified insert showing fibroblasts laying down collagen fibres.

Two patterns of fibrosis were identified in the mice; F1, which is pericellular and located around the central vein, primarily in acinus zone 3; and the more severe F3, which is pericellular with bridging, connecting the central veins (Figure 4a). Interestingly, and particularly apparent for F3, fibrosis is in the early stages as indicated by fibroblasts (stained green by the stain fast green) actively depositing collagen fibres (stained red by picrosirius red; see magnification of Figure 4b). The frequency of the patterns of fibrosis by severity is presented in Table 1.

#### 2.3.3. Effect on Liver Enzymes and Circulating Lipid Levels

Chronic feeding with a WD subsequently changes liver enzyme levels, indicative of hepatic perturbations [38]. Therefore, we examined the 2-week treatment impact of cannabinoids, both as monotherapy and in combination, on plasma liver enzymes, alanine aminotransferase (ALT) and aspartate aminotransferase (AST). Circulating lipid biomarker levels such as triglycerides (TG), total cholesterol (CHO), HDL-Cholesterol (HDL-C), and LDL-Cholesterol (LDL-C) were also estimated in plasma samples. Furthermore, levels of hs-CRP, albumin and creatinine were assessed as safety biomarkers.

**Figure 5:**
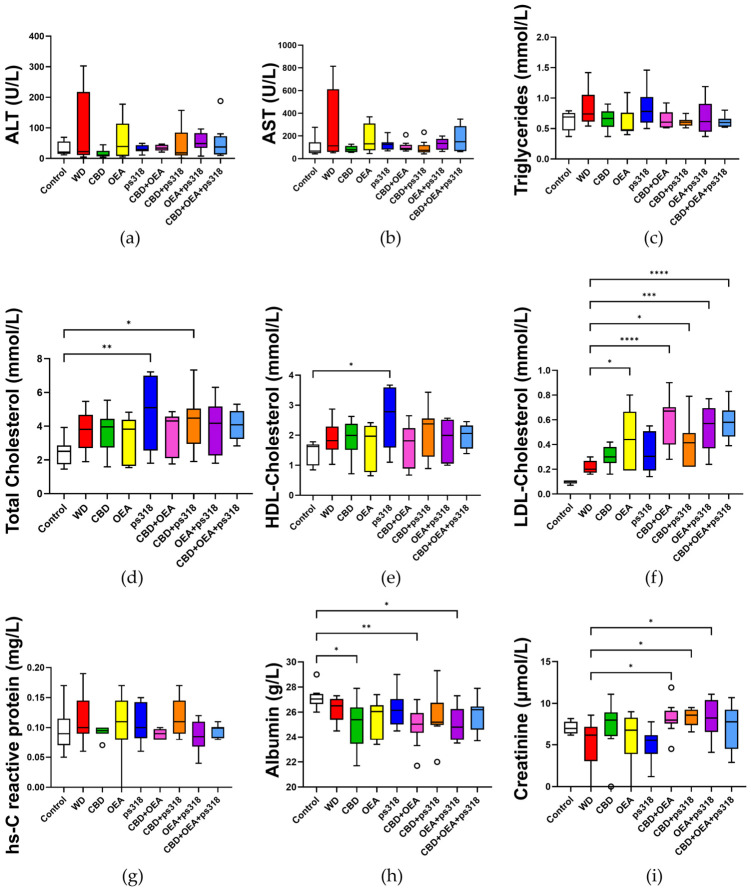
Key liver and lipid health biochemical markers quantified from serum at week 14: (**a**) ALT, alanine aminotransferase; (**b**) AST, aspartate aminotransferase; (**c**) triglycerides; (**d**) total cholesterol; (**e**) HDL, high-density lipoprotein cholesterol; (**f**) LDL, low-density lipoprotein cholesterol; (**g**) hs-C reactive protein; (**h**) albumin; (**i**) creatinine. All data were analysed using non-parametric method (see Section 4.6 for details). Abbreviations: * *p* < 0.05; ** *p* < 0.01; *** *p* < 0.001 and **** *p* < 0.0001; WD, Western-style fast-food diet. Circle indicates outliers and statistical comparisons were performed relative to the WD, except for total cholesterol and albumin.

WD mice exhibited increased ALT levels (Figure 5a) compared to normal chow (95.33 ± 40.25 vs. 30.53 ± 6.69 U/L). Similarly, monotherapy treatment of CBD (16.33 ± 4.36 U/L), OEA (59.11 ± 19.93 U/L), and compound ps318 (31.24 ± 4.36 U/L) showed a trend toward reducing ALT levels in plasma. Interestingly, CBD as a monotherapy showed the greatest reduction, although statistical significance was not achieved. The combination of CBD and OEA (34.68 ± 3.38 U/L) reduced ALT levels when compared to WD, as did treatment of CBD and compound ps318 (46.43 ± 17.35 U/L). The triple combination of CBD, OEA and compound ps318 (34.81 ± 9.54 U/L) and the combination of OEA and compound ps318 (52.95 ± 8.84) also reduced plasma ALT levels.

Similarly, AST levels (Figure 5b) showed an upward trend in mice fed the WD (282.2 ± 103.80 U/L), when compared to controls (10.3.4 ± 25.72 U/L). Monotherapy of CBD (85.87 ± 9.13 U/L), OEA (179.6 ± 39.81 U/L), and compound ps318 (121.9 ± 16.71 U/L) showed reductions in AST levels. The combination of CBD with OEA (91.70 ± 7.82 U/L) and with compound ps318 (95.18 ± 19.93 U/L), and OEA and compound ps318 (129.6 ± 15.41 U/L), and the triple combination (175.1 ± 36.48 U/L) showed non-significant trends towards reducing AST levels.

Cannabinoid treatment effects on circulating lipid profiles are presented in Figure 5c,d. Mice fed with WD exhibited a trend toward elevated CHO (3.66 ± 0.39 vs. 2.32 ± 0.19 mmol/L) and TG (0.76 ± 0.07 vs. 0.62 ± 0.05 mmol/L) concentrations relative to the normal diet control group. However, despite these diet-induced increases, none of the cannabinoid interventions, either as monotherapies or combinatorial regimens, produced a statistically significant reduction in circulating TG or CHO levels.

Circulating HDL-C (Figure 5e) and LDL-C (Figure 5f) levels were also analysed, revealing underlying changes to systemic metabolic health. Mice fed on a WD (1.90 ± 0.20 mmol/L) exhibited non-significant upward trend in HDL-C levels compared to the normal diet controls (1.43 ± 0.12 mmol/L) mice. Compared to the WD, CBD (1.88 ± 0.21 mmol/L) and OEA (1.63 ± 0.25 mmol/L) alone showed a downward trend in HDL-C levels. Similarly, combinations of CBD with OEA (1.67 ± 0.21), OEA with compound ps318 (1.82 ± 0.22 mmol/L), and triple combination (1.98 ± 0.13 mmol/L) groups showed similar trend. However, LDL-C results showed highly statistically significant changes between control (0.10 ± 0.00 mmol/L) and WD (0.22 ± 0.02 mmol/L, *p* < 0.0042). All monotherapy groups had no statistically significant changes in LDL-C levels, although all three groups showed an upwards trend when compared to both control and WD. Compared to WD, combination treatment of CBD and OEA drastically increased LDL-C levels (0.22 ± 0.02 vs. 0.60 ± 0.06 mmol/L, *p* < 0.0001), while combination treatment of CBD with compound ps318 also showed significant changes (0.22 ± 0.02 vs. 0.37 ± 0.04 mmol/L, *p* < 0.0493). Similarly, combination treatment of OEA and compound ps318 also increased LDL-C levels (0.54 ± 0.06, *p* < 0.0005) and triple combination treatment showed significant increases in serum LDL-C (0.59 ± 0.04, *p* < 0.0001).

Next, serum hs-C reactive protein (hs-CRP) (Figure 5g) was also measured as an identifier for chronic low-grade inflammation. Compared to the control (0.09 ± 0.01 mg/L) arm, the WD group (0.12 ± 0.01 mg/L) showed an upward trend in serum hs-CRP, signifying an increase in inflammation. CBD, OEA and compound ps318 monotherapy groups showed a downward trend in serum hs-CRP (0.09 ± 0.01, 0.10 ± 0.18, 0.11 ± 0.01 mg/L, respectively). The combination of CBD and OEA (0.10 ± 0.01) also showed similar downward trends; however, interestingly, the combination of CBD and compound ps318 (0.15 ± 0.03) showed an upward trend in serum hs-CRP. The combination of OEA and compound ps318 (0.08 ± 0.01) showed the greatest downward trend; however, this result was still not statistically significant. The triple combination of CBD, OEA and compound ps318 (0.11 ± 0.01) also showed a downward trend in comparison to WD.

Serum albumin (Figure 5h) was measured, with the normal chow group having an average of 27.16 ± 0.31 g/L. The WD group showed a slight decrease at 26.21 ± 0.32 g/L. Monotherapy of CBD (25.01 ± 0.60 g/L), OEA (25.48 ± 0.46 g/L) and compound ps318 (26.19 ± 0.44 g/L) also decreased serum albumin when compared to WD. When compared to normal controls, the treatment groups involving CBD in combination with OEA (24.93 ± 0.48 g/L, *p* < 0.025), as well as the combination treatment of OEA and compound ps318 (25.04 ± 0.44 g/L, *p* < 0.0132), were significantly different. Interestingly, the triple combination treatment did not yield statistically significant results (25.71 ± 0.43 g/L).

Serum creatinine (Figure 5i) in normal controls averaged around 7.09 ± 0.25 μmol/L, as compared to 5.2 ± 0.97 μmol/L. Monotherapy of CBD (7.40 ± 0.96 μmol/L) and OEA (5.91 ± 0.89 μmol/L) increased serum creatinine. Compound ps318 (4.98 ± 0.63 μmol/L) reduced serum creatinine when compared to controls. Combination treatment of CBD and OEA significantly increased serum creatinine levels when compared to WD, 8.21 ± 0.59 μmol/L, *p* < 0.0344, as did combination treatment of CBD and compound ps318 (8.31 ± 0.35 μmol/L, *p* < 0.0325). Combination of OEA and compound ps318 also significantly increased serum creatinine (8.24 ± 0.72 μmol/L, *p* < 0.0319); however, the triple combination of CBD, OEA and compound ps318 did not (7.12 ± 0.88 μmol/L).

#### 2.3.4. Effect on GPR55, GPR119, FAAH, and SREBP-1c Expression

**Figure 6:**
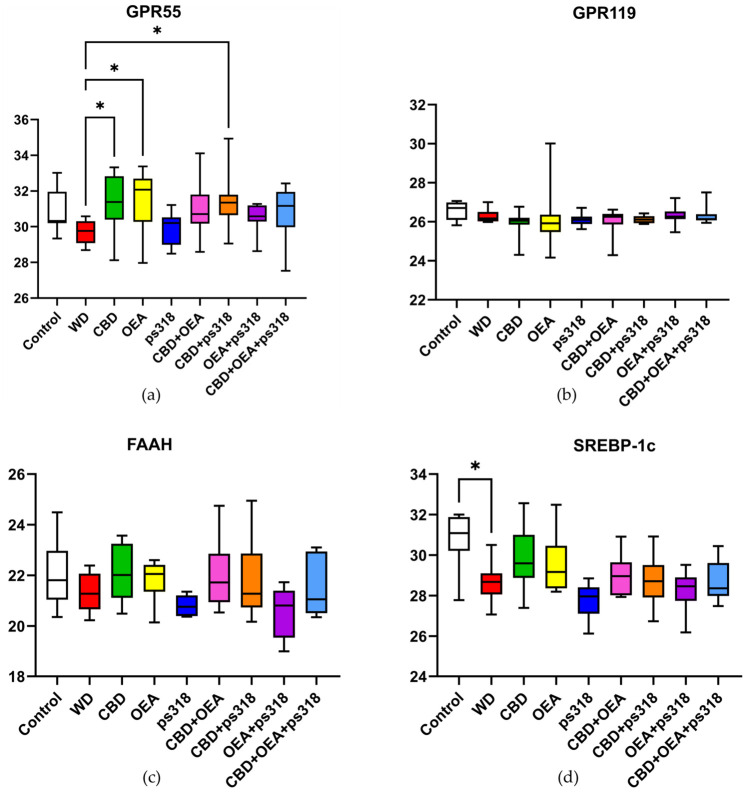
GPR55 (**a**), GPR119 (**b**), FAAH (**c**), and SREBP-1c (**d**) gene expression following 14-day cannabinoid treatment assessed by qPCR. Raw quantification cycle (Cq) values for hepatic expression across experimental groups are presented as a box-and-whisker plot (median with IQR), where lower Cq values indicate higher transcript abundance. Relative gene expression was calculated from Cq values using comparative quantification and expressed as fold change relative to the WD group. Statistical comparisons were performed against the WD group, with significance indicated as * = *p* < 0.05 and. Abbreviations: WD, Western-style fast-food diet.

We examined the effects of a 2-week cannabinoid treatment, administered alone or in combination, on the expression of metabolic and endocannabinoid-related genes, including GPR55, GPR119, fatty acid amide hydrolase (FAAH), and sterol regulatory element-binding protein-1c (SREBP-1c) (Figure 6). Raw Cq values for hepatic GPR55 across experimental groups are shown as box-and-whisker plots (median and IQR), where lower Cq values reflect higher transcript abundance. Hepatic GPR55 expression was elevated in the WD group, consistent with previous findings reported by Fondevila et al. [14] (Figure 6a). Cannabinoid treatment showed an overall trend toward reduced GPR55 expression, except for ps318 administered as monotherapy or in combination with OEA. CBD monotherapy significantly reduced GPR55 expression (~2.6-fold decrease, *p* < 0.01), as did OEA monotherapy (*p* < 0.05). Similarly, the combination of CBD with ps318 significantly decreased GPR55 expression (~2.6-fold, *p* < 0.01). These findings suggest that cannabinoid treatment, particularly with the GPR55 antagonist CBD, reduces hepatic GPR55 expression. Hepatic expression of GPR119 remained largely unchanged across both the WD and treatment groups (Figure 6b). FAAH, a key enzyme in endocannabinoid degradation, serves as an indirect marker of endocannabinoid tone. No statistically significant differences were observed between groups (Figure 6c). Hepatic SREBP-1c expression was measured as an indicator of lipogenic activity. A significant increase in SREBP-1c expression was observed in the WD group compared with normal controls (3.55-fold increase, *p* < 0.05), consistent with previous reports linking SREBP-1c upregulation to metabolic dysfunction. Cannabinoid treatment did not result in any statistically significant changes in SREBP-1c expression (Figure 6d).

## 3. Discussion

Targeting the eCB has emerged as a novel therapeutic strategy for MASLD/MASH and related metabolic disorders. Dysregulation of the eCB has been implicated in the pathogenesis of MASH through its control of appetite, lipid metabolism, blood glucose regulation, inflammation and fibrogenesis. Previous attempts at drug development targeting the canonical cannabinoid receptors CB1 and CB2 receptors have been widely unsuccessful, due to their unwanted off-target effects and relatively low expression in hepatocytes. As such, these impediments have driven drug discovery away from direct CB1/CB2 receptor modulation and toward the broader, more nuanced signalling network of the eCB [34,39]. Increasingly, GPCRs have been recognised as key modulators of metabolic activity and as highly responsive therapeutic targets [33,40]. Of particular interest, GPR55 and GPR119 have been found to be therapeutic targets of interest in MASH [41]. Activation of the LPI/GPR55 axis has been associated with pro-inflammatory and lipogenic signalling pathways [14], while activation of GPR119 has shown a hepatoprotective effect [15,42]. Hence by leveraging the agonist/antagonistic properties of cannabinoids to target the LPI/GPR55 axis and GPR119 receptors in both the gut and liver, a safer physiological intervention for MASH could be achieved.

CBD, a negative modulator of GPR55, has been shown previously to reduce hepatic lipid accumulation and fibrosis in preclinical models, indicating its potential to be included in a combination therapy [16]. Furthermore, the microbiota-produced OEA has been shown to activate GPR119, modulating energy homeostasis and having potent anorectic properties [43]. Compound ps318, a small-molecule agonist used at a suboptimal dose in this study, is a gut-oriented GPR119 agonist stimulating endogenous GLP-1 secretion from intestinal L-cells [29] and reported to have hepatoprotective activity in combination with sitagliptin [44,45]. Previous works have reported the synthesis and preliminary pharmacology of compound ps318, alongside an investigation into the acute effects in a model of diabetes [30]. This present study aimed to present a novel investigation into the effects of combination therapy using these cannabinoids to evaluate their efficacy in WD-fed mice. We investigated the effects of these cannabinoids on body weight, fasting blood glucose, hepatic health and blood biochemistry. Given that the WD for mice, high in fructose and fat, has been known to induce fatty liver disease and hepatocyte perturbations, we used this model to determine the efficacy of pharmacological treatment using cannabinoids. Mice were fed a WD for 12 weeks to develop obesity and MASH, with the associated insulin resistance [46,47].

### 3.1. Liver Steatosis and Ballooning, as Well as General Liver Condition

The activation of the LPI/GPR55 axis has been implicated in the progression of NAFLD, a term previously used to describe fatty liver disease [14]. The LPI/GPR55 axis acts as a pathogenic pathway for MASH, despite systemic activation of GPR55 exhibiting beneficial physiological roles [48,49,50]. That is, the downstream effects caused by the LPI/GPR55 axis skew the signalling pathway towards a pro-inflammatory and pro-lipogenic phenotype. The inconsistent presence of fibrosis in mice from the positive disease group was expected because this model did not target fibrosis (i.e., choice of diet (low cholesterol) and duration (12 weeks)); it was designed to target early features of MASH, particularly steatosis, hepatocyte ballooning and inflammation. Therefore, a lack of significance or trend between the treatments for the fibrosis was not unexpected.

Here, we hypothesised that CBD, an antagonist of GPR55, would improve liver conditions. At first glance, CBD, when compared to WD, did not significantly improve the liver-to-body-weight ratio. However, we can see that CBD did exert its hepatoprotective effects, evident from ALT and AST levels. This is concurrent with previously published works [51,52]. Furthermore, we observed that CBD monotherapy also alleviates hepatocyte ballooning. This follows published literature, showing that CBD has been established to combat oxidative stress in cells, primarily by reducing the production of reactive oxygen species [53,54].

OEA, a bioactive lipid synthesised in the gastrointestinal tract, has several distinctive homeostatic properties, including lipolysis, fatty acid oxidation, and anti-inflammatory properties [27,55]. OEA also acts as a GPR119 agonist, with evidence suggesting that intraperitoneal injection of OEA reduced feed intake and prevented steatohepatitis. [56,57] Furthermore, activation of GPR119 in hepatocytes was found to potently reduce hepatic lipid content, ALT and AST levels [58]. As such, we hoped to reproduce the same results in our present study, with even greater effect when combined with another GPR119 agonist—compound ps318—and a GPR55 antagonist. Monotherapy of OEA in our study showed significant reductions in the liver-to-body-weight ratio, as well as potently inducing weight loss in mice.

In the current study, the GPR119 agonist compound ps318 in a 14-day experimental model of MASH did not reduce the liver-to-body-weight ratio, weight loss, or fasting blood glucose when administered by itself. However, co-therapy of OEA and ps318 did significantly reduce weight, improving the liver-to-body-weight ratio and reducing insulin resistance. This phenomenon can be linked to previously seen results suggesting that when compound ps318 was administered in addition to DPP-IV inhibitor sitagliptin, significant improvements in metabolic health were observed [30]. One possible mechanism of action is that the gut-oriented ps318 has limited systemic bioavailability; however, its GLP-1-mediated effect is enhanced in the presence of a DPP-IV inhibitor [30].

Interestingly, when combined with the GPR55 antagonist CBD, the previously seen improvements in the liver-to-body-weight ratio were negated. However, we observed that there were improvements in hepatic ballooning, possibly suggesting that CBD-mediated GPR55 antagonism together with GPR119/incretin-axis activation promotes hepatoprotection via the gut-liver axis. CBD has demonstrated hepatoprotective effects, alleviating steatosis, inflammation and fibrosis in preclinical models [59,60,61]. On the other hand, the GPR119/incretin axis has also been established, through stimulating the release of GLP-1, as improving metabolic parameters [15,62]. As such, this evidence collectively suggests that both arms of GPR55 antagonism and the GPR119/incretin axis activation have support. Our study is the first study to examine the effects of this synergy, as to date, no study has directly tested combined CBD-mediated GPR55 antagonism alongside GPR119 activation in a liver disease model.

An important observation in this study was the increase in circulating LDL-C levels in several combination treatment groups. While this may initially appear adverse, it is mechanistically consistent with enhanced lipid mobilisation and export. In metabolic liver disease, hepatic steatosis reflects an imbalance between lipid influx, oxidation, and export, often characterised by impaired VLDL secretion and intracellular lipid trapping. Pharmacological interventions that reduce hepatic lipid accumulation frequently restore this balance by promoting lipid efflux. Therefore, the increase in LDL-C observed in our study likely reflects enhanced hepatic lipid export associated with reduced steatosis. This interpretation is further supported by concurrent reductions in liver weight, steatosis, and hepatocyte ballooning in cannabinoid-based treatment groups. This phenomenon of lipid redistribution, whereby intrahepatic lipid stores are mobilised into the circulation, has been described in metabolic interventions targeting hepatic fat and may represent an acute but necessary phase of metabolic normalisation [63,64]. Nonetheless, the long-term implications of sustained LDL-C elevation remain unclear and warrant further investigation to determine whether this compensatory rise resolves or contributes to increased cardiometabolic risk.

Furthermore, elevations in serum creatinine observed in select combination treatment groups may reflect acute changes in renal handling or systemic metabolic stress. Given the short duration of this study, these findings should be interpreted with caution. Long-term follow-up studies are needed to determine whether these changes represent transient metabolic adaptations or are indicative of underlying toxicity.

### 3.2. Blood Glucose

WD-fed mice developed obesity and subsequently reduced capacity to regulate blood glucose levels. Here, we see that CBD exhibited antihyperglycemic effects. CBD interacts with multiple receptors and is reported to interact with Transient Receptor Potential cation channel subfamily V member 1 (TRPV1), a part of the eCB [52]. However, our results varied from previously published literature, which found that intraperitoneal administration did not result in statistically significant differences in changes to blood glucose [52,65]. The authors suggest that oral administration of CBD at 50 mg/kg, a dose akin to our study, provided the greatest change to blood glucose. An explanation in line with this observation may be that, due to the lipophilic nature of CBD, absorption through the GI tract may be more efficient [66,67]. Alternatively, we chose to administer CBD intraperitoneally due to the limited oral bioavailability (around 6%) [68]. The glucose-lowering effect of OEA was also pronounced in our study, showing that mice fed a WD to induce insulin resistance showed marked improvements in their fasting blood glucose levels, aligning with previously known activity of GPR119 [69,70]. Also, OEA is able to activate PPAR-α, improving insulin sensitivity [23].

In combination, we noted an even greater glucose-lowering effect when CBD was co-administered with OEA, further highlighting the high therapeutic potential of modulating the eCB. When administered with ps318, we observed a statistically significant reduction in fasting blood glucose, albeit not as drastic as other combinations. Again, this may relate to the limited bioavailability and gut-oriented nature of compound ps318. Combination treatment using all three cannabinoids, CBD, OEA and ps318, showed the greatest effect on reducing fasting blood glucose, suggesting the synergistic properties of co-administration.

### 3.3. Body Weight and Feed Intake

As previously mentioned, both CBD and OEA induced significant weight loss, and this effect was further enhanced when the compounds were combined. Co-therapy of CBD and OEA drastically increased weight loss, and unsurprisingly, the combination of all three cannabinoids resulted in the greatest weight loss. OEA is able to act independent of the eCB, affecting sensory afferents that signal satiety to the brain [71]. Moreover, OEA is a potent activator of PPAR-α, a key regulator of fatty acid metabolism and ketogenesis [72]. Interestingly, when CBD was administered as a monotherapy, we saw the greatest amount of reduction in feed intake. Contrary to our hypothesis, OEA did not significantly reduce feed intake.

### 3.4. Modulation of Hepatic Gene Expression

Our treatments decrease GPR55 expression compared to the WD and support the conclusion that the metabolic and hepatic improvements observed in our model are associated with a downregulation of GPR55-related signalling within the eCB. This suggests that, under Western diet-induced metabolic stress, GPR55 may be upregulated or contribute to a maladaptive state, and that its suppression is linked to beneficial outcomes. Cannabidiol, OEA, and ps318, particularly in combination, exert their effects at least in part by normalising or dampening GPR55 signalling, which may be involved in promoting inflammation, metabolic dysregulation, or liver injury in this context. This aligns with the broader concept that targeting the extended endocannabinoid system can restore metabolic balance. However, the conclusion should remain cautious. The data demonstrate an association between reduced GPR55 expression and improved metabolic and hepatic parameters, but they do not establish causality. Therefore, downregulation of GPR55 is a potential contributing mechanism underlying the therapeutic effects, and further studies are needed to clarify its functional role. While the absence of an endogenous reference gene represents a methodological constraint, it reflects the lack of a validated invariant control under these conditions; future studies should aim to validate stable reference genes and GPR55 expression changes. Overall, our conclusion is that beneficial outcomes correlate with suppression of GPR55-associated signalling, reinforcing the therapeutic relevance of modulating the eCB in metabolic liver disease.

### 3.5. Limitations and Translational Considerations

It is important to acknowledge the inherent variability in diet-induced disease models, which may complicate the interpretation of treatment effects. Previous studies have documented substantial inter-subject differences in weight gain and metabolic outcomes in rodents exposed to identical obesogenic diets [73,74,75]. Moreover, even genetically homogeneous inbred strains of mice can exhibit differences in intrinsic physical activity and food intake, thereby influencing susceptibility to obesity and associated metabolic dysfunction. In our study, mice with lower baseline body weight followed distinct trajectories in body mass and adiposity compared with heavier counterparts. This heterogeneity represents a known limitation of diet-induced models, potentially masking intervention effects and reducing reproducibility. In addition, the relatively short treatment duration (14 days) limits the ability to assess long-term outcomes, including fibrosis progression or reversal, and overall safety in the context of a chronic condition. Accordingly, the present findings should be interpreted as reflecting acute metabolic and hepatic responses rather than sustained disease modification. Mechanistically, while the study was designed to explore the therapeutic potential of targeting the endocannabinoid system, it does not include genetic or pharmacological validation (e.g., receptor-specific knockout models or pathway inhibitors). This represents an important limitation, and future studies incorporating such approaches will be necessary to delineate the specific signalling pathways and potential synergistic interactions underlying the observed effects. The current work should therefore be considered as providing preliminary evidence supporting this hypothesis. Furthermore, comparisons with established or emerging standard-of-care therapies, such as GLP-1 receptor agonists (e.g., semaglutide), were not included. These agents act through distinct mechanisms and were beyond the scope of this initial combinatorial cannabinoid-focused investigation; however, such comparisons will be important in future studies to better contextualise efficacy. The study also lacks dose–response and toxicity assessments, which are essential for evaluating therapeutic windows, safety profiles, and translational potential. These aspects represent key priorities for subsequent investigations. Finally, the exclusive use of a murine model limits direct clinical translation. No in vitro or human validation data (e.g., primary human hepatocytes or patient-derived samples) were included. Nonetheless, the observed changes in body weight, glycaemic control, liver enzyme profiles, and histopathological features represent clinically relevant endpoints commonly used in metabolic liver disease research. Moreover, the use of a Western diet model enhances translational relevance by recapitulating key aspects of human metabolic risk factors.

Collectively, these limitations highlight the need for future studies incorporating longer treatment durations, mechanistic validation, comparative pharmacology, and human-relevant models to further define the therapeutic potential of endocannabinoid modulation.

## 4. Materials and Methods

### 4.1. Drugs, Chemicals, Reagents and Animal Diet

Carboxymethyl cellulose (CMC), Tween-80^®^, dimethyl sulfoxide (DMSO) and sterile saline solution were purchased from Sigma-Aldrich, St Louis, MO, USA. A blood glucometer with compatible strips (Accu-check Performa; Roche Ltd., Basel, Switzerland) was locally sourced from a pharmacy. CBD was obtained from Sigma Aldrich, OEA from Frau Pharma and the investigated compound ps318 was synthesised in-house as previously described [29]. Biochemical markers were assessed by PathWest Laboratory Medicine Western Australia (Fiona Stanley Hospital). The normal rodent chow diet (SF00-100) and Western-style fast-food diet (SF-21-155) were formulated locally by Specialty Feeds, Perth, WA, Australia.

### 4.2. Experimental Animals, Husbandry, and Ethical Considerations

Five-week-old healthy male C57BL6/J mice were procured from Ozgene (formerly Animal Resources Centre), Perth, WA, Australia. The mice were housed and acclimatised at Curtin University’s animal facility for one week. After acclimatisation, the animals were then divided into respective treatment groups at random. A subset of animals (n = 10) was fed a normal chow diet and kept as a control group. The rest of the mice received the WD to induce MASLD. Animals were fed a Western-style diet consisting of SF21-155 (20% fructose, caseinate-modified formulation of SF03-020; Specialty Feeds, Perth, WA, Australia), in combination with a Meat-Free Rat and Mouse Maintenance Diet (Specialty Feeds, Perth, WA, Australia). The diet is enriched in fructose and modified protein sources to model features of a Western dietary pattern. Throughout the study, all animals had ad libitum access to food and drinking water. Mice were housed in ventilated cages under controlled environmental conditions, including a temperature of 22 ± 2 °C, humidity of 55 ± 5%, and a 12:12 h light-to-dark cycle. Animal use was in accordance with the Australian Code for the Care and Use of Animals for Scientific Purposes 8th Edition 2013 (Updated 2021). All animal procedures were performed in accordance with institutional animal care guidelines and conducted according to the animal ethics committee-approved protocols. Animal ethics approval number ARE2023-21 was filed and obtained with Curtin University, Perth, WA, Australia.

### 4.3. Study Design, Treatment Groups and Drug Formulations

Treatments (monotherapy and combination therapy) were administered to mice following twelve weeks of WD. Mice were randomly assigned to treatment groups, with each treatment group consisting of 10 (n = 10) animals. A total of n = 90 animals were used for this study. Sample size was determined based on similar previous experiments and calculated using power analysis, assuming a significance of α = 0.05 and statistical power (1 − β) = 0.8, in line with standard recommendations for preclinical animal studies. Following completion of diet treatment, the animals were then treated with pharmacological agents for fourteen days. CBD (50 mg/kg) and OEA (50 mg/kg) were administered intraperitoneally; ps318 (50 mg/kg) was administered orally. Compound ps318 was administered in a suboptimal dose as compared to previous studies [30], due to the addition of further treatment compounds. The reason for this exploratory dosage was to determine the additive effects of compound ps318 in combination with other pharmacological agents, without exceeding tolerability thresholds. This approach allows assessment of potential synergistic interactions between GPR119 agonism and GPR55 antagonism. Placebo control animals (n = 10) were administered similarly with vehicle. Outcome assessors and data analysts were blinded to treatment allocation to minimise observer and analytical bias in accordance with established experimental design guidelines.

### 4.4. Pharmacological Assessments and Sample Collection

Body weight and food intake were measured three times weekly. Energy consumption was measured by multiplying feed intake by specifications for feed (maintenance diet: 12.8 kJ/g; Western diet: 19.7 kJ/g). Fasting blood glucose was recorded on the final day of the study, after fasting for five hours. Endpoint blood collection was completed by cardiac puncture, while mice were under anaesthesia. Blood samples were collected in K_2_EDTA-coated tubes containing a DPP-IV inhibitor cocktail (EMD Millipore, DPP4-010, Burlington, MA, USA) to prevent enzymatic degradation of metabolic hormonal peptides. Plasma was isolated by centrifugation at 8000 rpm for 10 min at 4 °C and stored at −80 °C for further analysis.

Mice were euthanized under isoflurane anaesthesia (Piramal Pharma Ltd., Mumbai, India) by cervical dislocation. Following dissection, the liver, small intestine and caecum were collected and snap frozen in liquid nitrogen. Livers were weighed prior to snap freezing to calculate the liver-to-body-weight ratio. Samples were stored at −80 °C, with a portion of liver tissue stored in 10% neutral buffered formalin solution for subsequent histological examinations.

### 4.5. Liver Histological Investigations

Microscopic histopathological examinations were conducted on liver tissues fixed in formalin. Tissues were embedded in paraffin blocks and sectioned into 4 μm slices using a microtome (Leica Biosystems Nussloch GmbH, Nußloch, Germany). Tissue sections were then mounted on glass slides and stained with haematoxylin and eosin (H&E; Hurstchem Laboratory Chemicals, Forrestdale, WA, Australia). Digital images were captured at 20× magnification using the digital slide scanner Zeiss AxioScan Z.1 with Zen v3.8.3 software (Carl Zeiss GmbH, Oberkochen, Germany). Histopathological evaluation of MASH was based on the established histological features of steatosis, inflammation and hepatocyte damage (ballooning) [76,77], with the following exceptions to better capture the histological outcomes of pharmacological treatment. Steatosis was quantified digitally as a percent of total area (H&E section) by selecting ten representative regions of interest (ROI). The ROIs were batch-analysed with an Intellesis model (Carl Zeiss GmbH, Germany) trained on representative images to identify total steatosis. Inflammation was semi-quantitatively evaluated, blinded, by averaging the number of inflammatory foci in three representative ROIs of H&E sections (3 × 1.5 mm^2^). Identification of inflammatory foci consisted of ≥5 immune cells in a cluster: not in a row [76]. Inflammation was assessed by hepatocellular ballooning, scored as the average across three representative regions of interest (ROIs) and evaluated in a blinded manner. Hepatocyte ballooning score was expanded (0–4) to more accurately capture the extent of hepatocyte damage in this model: 0, none to few scattered ballooned hepatocytes that are often seen in healthy animals; 1, low (several scattered individual ballooned hepatocytes or isolated, small foci); 2, moderate (moderate to many scattered individual ballooned hepatocytes and/or several foci); 3, high (many ballooned hepatocytes, mostly grouped as several to many foci affecting large regions); 4, severe (many interconnected foci of ballooned hepatocytes throughout tissue). For the evaluation of fibrosis, sections were dehydrated and stained with 0.04% (*w/v*) picrosirius red (Merck Life Science Pty Ltd., Bayswater, VIC, Australia) in saturated picric acid (Merck Life Science) for 30 min followed by counterstaining with 0.1% fast green (Merck Life Science) for 10 min. Sections were then dehydrated and imaged as for the H&E sections. Fibrosis staging was performed at 100× magnification for the entire section according to the following categories: F0, no fibrosis; F1, mild, zone 3 (surrounding central vein) perisinusoidal/pericellular fibrosis; F2, moderate, zone 3 + portal fibrosis (zone 1), no bridging; F3, severe, bridging fibrosis; F4, cirrhosis [77].

### 4.6. RT–qPCR (Reverse Transcription Quantitative PCR)

Complementary DNA (cDNA) synthesis was performed using the ABScript II cDNA First Strand Synthesis Kit (ABClonal, Woburn, MA, USA), according to the manufacturer’s instructions (Version M17H04v2.3). Briefly, 500 ng of total RNA was combined with reverse transcription reagents using buffer, dNTPs, reverse transcriptase and a mixture of oligo(dT) and primers. The reaction was then incubated at room temperature for 5 min for primer annealing, followed by reverse transcription at 42 °C for 15 min and enzyme inactivation at 85 °C for 5 min. Synthesised cDNA was then diluted and stored at −20 °C. Total RNA was extracted from tissue samples and quantified to ensure equal input across all samples. cDNA was synthesised from equal amounts of RNA using standardised reverse transcription conditions. Gene expression was assessed using raw quantification cycle (Cq) values without normalisation to a single housekeeping gene. This approach was adopted due to evidence that commonly used reference genes may exhibit variable expression under metabolically perturbed conditions, such as Western diet-induced liver disease. To minimise technical variability, strict input normalisation was applied, with equal RNA quantities used for cDNA synthesis and identical reaction conditions maintained across all samples [78,79].

Under these conditions, Cq values were used for direct comparison of relative transcript abundance between groups. Statistical analyses were performed on Cq values to preserve linearity. While this approach does not account for potential global changes in total mRNA content, all samples demonstrated comparable RNA quality and were processed in parallel to ensure consistency. Quantitative real-time PCR (qPCR) was performed using SYBR Green chemistry on a real-time PCR detection system (Bio-Rad CFX). Reactions were prepared in a final volume of 10 μL containing SYBR Green master mix, cDNA template and gene-specific primers listed below:

Mouse GPR55:
Forward sequence: CTATCTACATGATCAACTTGGCTGTTT
Reverse sequence: TGTGGCAGGACCATCTTGAA
Amplicon: 201 bp
Mouse SREBP-1c:
Forward sequence: GGAGCCATGGATTGCACATT
Reverse sequence: GGCCCGGGAAGTCACTGT
Amplicon: 124 bp
Mouse FAAH:
Forward sequence: AGATTGAGATGTATCGCCAG
Reverse sequence: CTTCAGAATGTTGTCCCAC
Amplicon: 212 bp
Mouse GPR119:
Forward sequence: GGCTGATACCTTGATTGGCGTG
Reverse sequence: TGCCATCCGAAGGCTACACAAG
Amplicon: 110 bp

### 4.7. Statistical Methods

Graphical presentations, calculations and statistical analysis were performed using GraphPad Prism © version 10 (San Diego, CA, USA). The D’Agostino-Pearson test was used to evaluate if data had a normal distribution. If normally distributed, the data was analysed with the one-way ANOVA, followed by Tukey’s test for multiple comparisons and Dunnett’s test for comparing all groups to the control group. A significance level at the 95% confidence interval was considered statistically significant (*p* < 0.05). Results are expressed as mean ± standard error of the mean (SEM). Data that was not normally distributed was analysed with the non-parametric Kruskal–Wallis test and Dunn’s test was used for multiple comparisons. Outliers were identified and removed using the robust regression and outlier removal (ROUT) method with a false discovery rate (Q) of 5%.

## 5. Conclusions

To our knowledge, this is the first study to investigate the therapeutic effects of combination cannabinoid treatment in a mouse model of metabolic liver disease. Targeting the endocannabinoid system, even acute treatment markedly improved metabolic parameters, including significant weight loss, reduced fasting blood glucose, and improved liver condition. The triple cannabinoid combination produced the most pronounced effects, improving markers of hepatic injury and inflammation. Mechanistically, modulation of the LPI/GPR55 and GPR119/incretin axes highlights the therapeutic potential of targeting the gut–liver axis using small-molecule agonists and endogenous bioactive lipids.

## 6. Patents

MF is an inventor of a patent related to oleoyl-LPI mimetics (compound ps318), which is owned by Lipovexa SRL.

## Figures and Tables

**Table 1:** Shows the severity of the fibrosis and the frequency with which it occurred in the mice per treatment group. Abbreviations: WD, Western-style fast-food diet.

Treatment	Pericelluar Fibrosis (F1)	Pericellular Fibrosis w/Bridging (F3)	No Fibrosis
Control	0	0	10
WD	2	2	6
CBD	0	2	8
OEA	0	3	7
ps318	0	1	9
CBD+OEA	3	1	6
CBD+ps318	0	1	9
OEA+ps318	0	0	10
CBD+OEA+ps318	1	3	6

## Data Availability

The original contributions presented in this study are included in the article. Further inquiries can be directed to the corresponding author.

## Abbreviations

The following abbreviations are used in this manuscript:

ALT	Alanine aminotransferase
AST	Aspartate aminotransferase
CBD	Cannabidiol
CB1	Cannabinoid receptor-1
CB2	Cannabinoid receptor-2
CHO	Cholesterol
cDNA	Complementary DNA
DPP-IV	Dipeptidyl peptidase-4
eCB	Endocannabinoidome
FAAH	Fatty acid amide hydrolase
GLP-1	Glucagon-like peptide 1
GPR55	G-protein coupled receptor 55
GPR119	G-protein coupled receptor 119
H&E	Haematoxylin and Eosin
HCC	Hepatocellular carcinoma
HDL	High-density lipoprotein
LPI	L-α-lysophosphatidylinositol
MASLD	Metabolic dysfunction-associated steatotic liver disease
MASH	Metabolic-dysfunction associated steatohepatitis
OEA	Oleoylethanolamide
PPAR-α	Peroxisome proliferator-activated receptor alpha
qPCR	Quantitative polymerase chain reaction
ROIs	Regions of interest
SREBP-1c	Sterol regulatory element-binding protein-1c
TRPV1	Transient Receptor Potential Vanilloid 1
TG	Triglycerides
VLDL	Very-low-density lipoprotein
WD	Western fast-food diet
